# Case report: Clonal evolution analysis of a rare case of meningioma lung metastases identifies actionable alterations in matched longitudinal tumour samples

**DOI:** 10.3389/fonc.2024.1483126

**Published:** 2025-01-28

**Authors:** Nicola Cosgrove, Orla M. Fitzpatrick, Liam Grogan, Bryan T. Hennessy, Simon J. Furney, Sinead Toomey

**Affiliations:** ^1^ Genomic Oncology Research Group, Department of Physiology and Medical Physics, RCSI University of Medicine and Health Sciences, Dublin, Ireland; ^2^ Department of Medical Oncology, Beaumont Hospital, Dublin, Ireland; ^3^ Cancer Clinical Trials and Research Unit, Beaumont Hospital, Dublin, Ireland; ^4^ Medical Oncology Group, Department of Medicine, RCSI University of Medicine and Health Sciences, Dublin, Ireland

**Keywords:** meningioma, lung metastases, whole genome sequencing, targeted therapies, driver mutations

## Abstract

Metastatic meningioma is rare, occurring in less than 1% of patients, and very few case studies have been reported, in particular for those that have spread to the lungs. Here we describe a rare case of metastatic meningioma to the lungs. Following a discussion at a medical oncology multi-disciplinary team meeting, whole genome sequencing was requested in November 2021 and discussed at a neurosurgical molecular tumor board in June 2022. Sequencing was performed on matched longitudinal collected samples of the primary tumor resection, the re-excised recurrent tumor after adjuvant radiation therapy, the lung metastases before treatment with sunitinib, and one paired blood sample for tumor-normal analysis. Whole genome characterization and clonal evolution analysis confirmed neurofibromatosis 2 (NF2) gene loss as the main driver of this cancer. In the same cancer clone as NF2, we identified a BRCA2 (p.E51K) mutation was present in all tumors, which may represent a potential driver event, though evidence supporting this is currently limited. Although this mutation is predicted to potentially influence homologous recombination, its clinical relevance as a biomarker for PARP inhibition remains speculative and requires further investigation. We also noted a SETD2 (p.S1885N) mutation that was present only in the recurrent tumor which was identified as a predicted biomarker of response to WEE1 inhibition. There was a stepwise increase in tumor mutational burden (TMB) from the primary meningioma to lung metastases, suggesting this patient may have been a candidate for immunotherapy.

## 1 Introduction

Meningioma is the most common type of primary brain tumor (1) with most being slow-growing benign low-grade tumors treatable using surgery and adjuvant radiotherapy. Tumors are classified according to the World Health Organization (WHO) grading system: Grade 1, 2, or 3 (2). Compared to WHO Grade 1 tumors, high-grade tumors are less common, with WHO Grade 2 (atypical) malignant meningiomas occurring at an age-adjusted incidence rate of ~9.12% per year (1). These tumors are more likely to be invasive and recur locally following initial treatment in 30%-50% of all patients (1). Metastatic meningioma is rare, occurring in less than 1% of patients (3), with lung the most common site of metastasis (4). Due to their rarity, very few case studies have been reported. Genomic characterization of meningioma has overall improved our understanding of the underlying tumor biology and helped refine tumor classification and identify potential alterations for targeted therapy (5–12). Sequencing efforts have primarily focused on low-grade meningiomas, while characterization of high-grade and/or metastatic tumors remains relatively infrequent (7); however, integrated genomic analyses have highlighted key pathways, such as the co-mutation of SMARCB1 in atypical meningiomas (13). The loss of chromosome 22 is one of the most common genomic alterations that affect meningiomas (14). On this chromosome, inactivation of the tumor suppressor gene neurofibromatosis 2 (NF2), which encodes for the protein *Merlin*, is the main driver of 50% of meningiomas (9, 14). Compared to low-grade *Merlin*-intact tumors, NF2-altered tumors are more likely to be high-grade tumors with less favorable clinical outcomes (9). Here, we present a case report of metastatic meningioma to the lungs where longitudinal collected tumor samples underwent whole genome sequencing. The aim was to better understand the molecular drivers of this metastatic meningioma and identify any potentially actionable alterations in the lung metastases. At present, there are no standard treatments for metastatic meningioma (8) and, as such, comprehensive genomic profiling of such cases not only furthers our knowledge of this rare entity in cancer but also may guide personalized medicine decision-making.

## 2 Case description

### Presentation and history

**Figure 1:**
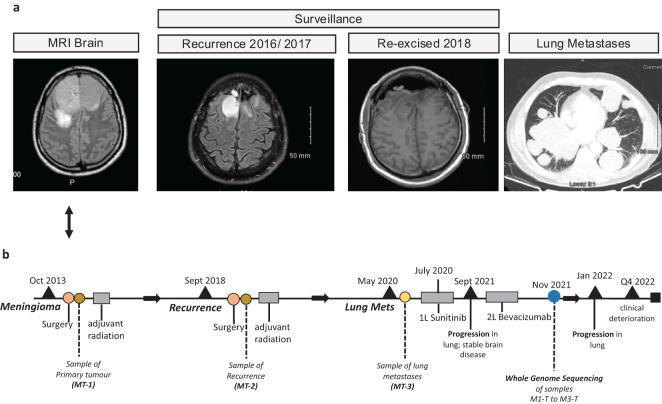
Case clinical history. **(A)** Images from magnetic resonance imaging (MRI) brain scans from the meningioma lung metastases case are presented here. **(B)** Graphical timeline plot summarizing the sample collection, treatment, and clinical event history for this case.

A 52-year-old man with an Eastern Cooperative Oncology Group (ECOG) performance status of 1 initially presented with an isolated episode of collapse in October 2013. Prior to this presentation, he and his family had noticed behavioral changes, worsening short-term memory, and headaches not relieved by simple analgesia. A brain MRI scan depicted a large bifrontal mass lesion (**Figure 1A**) which straddled the midline on either side of the interhemispheric fissure in the anterior cranial fossa, measuring 7cm in width and almost 6cm in the anteroposterior dimension, consistent with a meningioma. Due to his escalating symptoms, he underwent a resection. The resection was performed via a bi-coronal flap. The elevation of one flap intraoperatively was noted to be difficult due to adherence to the meningioma. The meningioma was enucleated and a frozen sample at the time of surgery confirmed a meningioma. The base of the tumor arose from the underside of the sagittal sinus. The anterior aspect adhered to the falx, and the ipsilateral layer of the falx and surrounding tissues underwent diathermy. A Simpson 2 resection was achieved. This resected specimen confirmed a WHO grade 2 meningioma with focal rhabdoid changes. The specimen was moderately cellular, with large, ovoid nuclei and centrally located nucleoli with frequent mitosis and copious amounts of eosinophilic cytoplasm. Some cells showed intracytoplasmic ill-defined hyaline structures consistent with rhabdoid change. The proliferation index as assessed by MIB-1 was elevated at approximately 10%-15%. Adjuvant radiation of 60Gy/30 fractions was completed following recovery from surgical resection and a discussion at a neurosurgical multidisciplinary team meeting. Following this, the patient began a surveillance program with MRI scans performed every 3 months initially, which demonstrated postoperative encephalomalacia and gliosis within both the anterior and parasagittal frontal lobes. The interval increased to 6 months until the end of 2016 when a brain MRI scan demonstrated a local recurrence (**Figure 1A**). This MRI brain scan with contrast that showed the recurrence of his disease depicted two areas of lobulated enhancing tissue within the resection cavity. However, it was also noted that there was surrounding T2/FLAIR hyperintensity within the frontal lobes, consistent with post-radiation change. During this period, the patient remained asymptomatic with an ECOG performance status of 1 and he continued to work. However, due to further progression over two subsequent MRI scans, which showed increasing areas of enhancement extending from the prior resection cavity to the right frontal horn resulting in a midline shift, and discussion with his neurosurgical team, he opted to proceed with re-resection in September 2018. A histological examination demonstrated a recurrence of the WHO grade 2 meningioma. The patient completed a further course of radiation therapy (54Gy/30fractions), and recovered well, returning to work following recovery from radiation. Microsatellite instability (MSI) testing of the specimen resected in 2018 confirmed a microsatellite stable (MSS) tumor. Further surveillance MRI was performed at 3-month intervals until May of 2020 when the patient presented with an episode of collapse associated with a 2-month history of grade 1 dyspnoea and dry cough. Diagnostic imaging with a CT scan of his thorax, abdomen, and pelvis with contrast performed during this admission demonstrated multiple bilateral pulmonary masses (**Figure 1A**). Bronchoscopy and biopsy of the pulmonary lesions confirmed metastatic meningioma.

The standard of care adjuvant therapy in high-grade or atypical meningiomas is radiation, however, in this case, referral for consideration of systemic treatment was made due to the recurrence after radiation of new and progressive systemic disease. In July 2020, the patient was referred to medical oncology where he was commenced on sunitinib based on data from other case reports (15). These case reports showed that, in a small exploratory cohort of 13 patients, sunitinib is active in patients with recurrent/atypical meningioma with a progression-free survival of 5.2 months (15). He remained on sunitinib for 14 months and tolerated it well, with regular disease monitoring including both CT and MRI. A repeat CT scan of the thorax, abdomen, and pelvis and a brain MRI scan performed after 14 months of treatment, identified progression of the disease in the thorax, but stable disease in the brain. At this point, the patient was switched to bevacizumab based on a systemic review carried out by Franke et al. (16) but his disease progressed further after 4 months of this treatment, with progression again isolated to the disease in the lungs.

Following a discussion at a medical oncology multi-disciplinary team meeting, whole genome sequencing (WGS) was requested in November 2021 and discussed at a neurosurgical molecular tumor board (MTB) in June 2022. Detailed descriptions of the WGS, mutation, and copy number variant analyses are supplied in the **Supplementary Material**. Unfortunately, due to significant clinical deterioration and presentation with multiple episodes of seizure over the months preceding the MTB, the patient was not administered any further lines of systemic anti-cancer treatment.

### Genomic characterization of meningioma lung metastases

WGS was performed on samples taken from the primary tumor resection (M1-T), the re-excised recurrent tumor (M2-T) after adjuvant radiation therapy, the lung metastases (M3-T) before treatment with sunitinib, and one paired blood sample for tumor-normal analysis (**Figure 1B**; **Supplementary Figure S1**). WGS data was used for genomic characterization of somatic single nucleotide variants (SNVs), insertions and deletions (InDels), and copy number alterations (SCNAs) across all tumor samples. We used the Bi et al.*’s* genomic sequencing of high-grade meningioma tumor samples (7) and Nassiri et al.*’s* molecular subgroups (11) as a reference for molecular classification and characterization of the meningioma tumors profiled here (**Supplementary Figure S2**).

**Figure 2:**
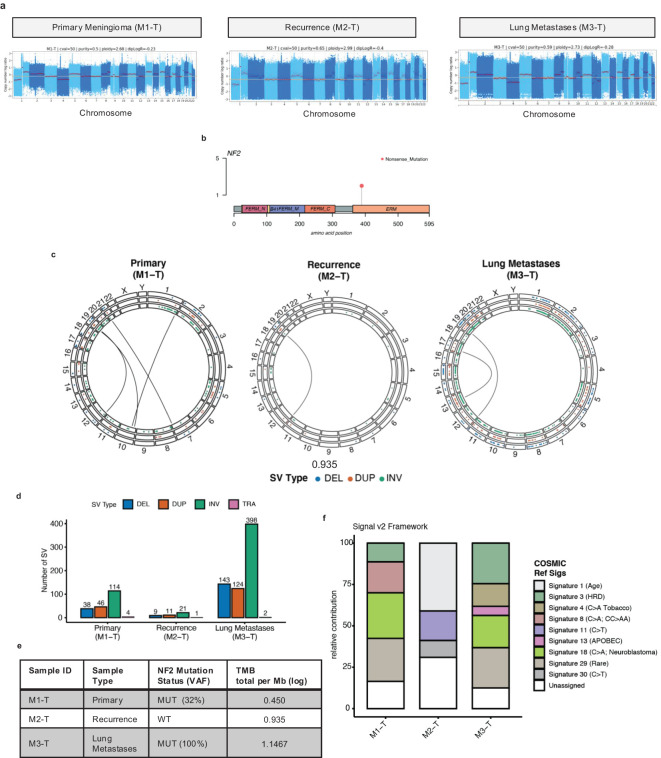
Genome-wide characterization of meningioma lung metastases tumor samples. **(A)** Somatic copy number log ratio (tumor over normal) coverage plots for the primary tumor (M1-T), recurrence (M2-T), and lung metastases (M3-T). The red horizontal line indicates a diploid log ratio reference. **(B)** Lolliplot of a recurrent missense mutation Q389* (orange dot) identified in the Merlin protein encoded by the NF2 gene from the WGS data **(C)** Circos plots of genome-wide chromosomal structural variation [DEL: deletions (blue dots); DUP: duplications (orange dots); INV: inversions (green dots); inter chromosomal translocations (TRA) indicated by black center line links] across all tumor samples. **(D)** Bar chart of the number of somatic SVs detected in each tumor sample with the colors indicating SV type [DEL (blue), DUP (orange), INV (green), TRA (pink)]. **(E)** Summary of the nonsynonymous TMB estimates [log total per megabase (Mb)] for tumor samples (M1-T, M2-T, M3-T). **(F)** Stacked bar chart of the relative contribution (0-100) of the COSMIC reference mutational signatures (Signature 1-30) detected in each tumor sample (left-right) using the Signal (v2) mutational signature profiling framework.

The genome-wide somatic copy number profiles were remarkably similar across all tumor sample timepoints (**Figure 2A**). Chromosome 22q loss encompassing the NF2 gene was present in all tumors along with SCNAs previously reported in Grade 2 and Grade 3 meningioma primary tumors including chr1p, chr6q, chr10, chr14q, chr18p, and q copy number loss. In the NF2 gene, a C>T nonsense (stop gained) mutation was detected in the primary tumor (M1-T) and lung metastases (M3-T) at variant allele frequency (VAF) estimates of 32% and 100% (**Figures 2B, C**). This suggests that in combination with chromosome 22q loss, biallelic NF2 inactivation occurred in these tumors. Structural variant calling identified a large number of intrachromosomal inversions in both the primary tumor (n=114) and lung metastases (n=398) (**Figures 2D, E**) with few interchromosomal translocations (TRAs) identified.

Overall, the SCNA profiles here are characteristic of higher-grade meningioma tumors and of the MG4 proliferative and hypermitotic tumor subtypes reported by Nassiri et al. and Choundary et al. respectively (11, 12). Next, the tumor mutational burden (TMB) was calculated as the total number of nonsynonymous somatic mutations divided by the exome region length in megabases (Mb) (**Figure 2F**). This analysis identified a twofold increase in TMB from 0.450 (log TMB/Mb) in the primary meningioma tumor to 0.935 (log TMB/Mb) in the recurrence and 1.1467 (log TMB/Mb) in the lung metastases. The primary and recurrence TMBs were in the same range as previously reported nonsynonymous TMB estimates for higher-grade, NF2-altered recurrent meningioma tumors (**Supplementary Figure S2**). Next, in order to investigate which mutagenic process may have led to a stepwise increase in TMB and chromosomal instability with disease progression, we performed a mutational signature analysis of somatic single nucleotide variants (**Figure 2F**; **Supplementary Figure S3**). Mutational signatures extracted from tumor samples were fitted to Catalogue Of Somatic Mutations In Cancer (COSMIC) reference signatures (Signatures 1-30). In both the primary meningioma and lung metastases we detected Signature 3 [associated with homologous repair deficiency (HRD)], Signature 18 (C>A; etiology unknown; found in neuroblastoma) and Signature 29 (Rare; C>A mutation in tobacco chewing). Signature 4, a C>A mutation pattern associated with exposure to tobacco smoking [this case is an ex-smoker (~25 pack years)] and APOBEC-associated Signature 13 were detected only in the lung metastases, with Signature 8 (C>A; found in medulloblastoma) found only in the primary meningioma. None of these signatures were detected in the recurrence, which was overall dominated by a C>T mutation context pattern associated with three mutational signatures: Signature 1 (ageing), Signature 11 (alkylating agents in glioblastoma and melanoma), and Signature 30 (BER deficiency). In the recurrence, we did detect a predicted loss of function driver mutation in SETD2, a reported DNA MMR regulator (17), however, the MSS classification given by MSI testing, performed by the molecular pathology team in 2018, was microsatellite stable.

**Figure 3:**
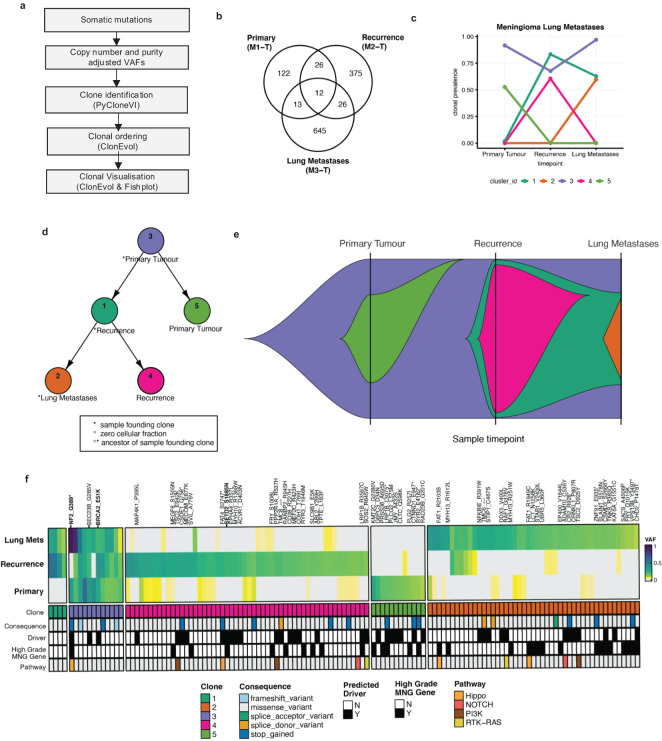
Clonal evolution in the meningioma lung metastases. **(A)** High-level graphical overview of clonal evolution analysis workflow. **(B)** Venn diagram of the nonsynonymous somatic mutation counts from all tumor samples. **(C)** Line plot of clonal prevalence (0-1.0) changes across all tumor sample timepoints (left to right). Five tumor clones [Clone #1 (dark green), #2 (orange), #3 (purple), #4 (pink), #5 (purple)] were identified using PyCloneVI. **(D)** Node tree plot shows the clonal ordering of the tumor clones from ClonEvol. **(E)** Fish plot shows a graphical representation of the clonal evolution across all tumor sample timepoints for the primary tumor (left), recurrence (middle), and lung metastases (right). **(F)** Heatmap of VAF (0-1.0) values for a subset of the total nonsynonymous somatic mutations for the primary meningioma (bottom), recurrence (middle), and lung metastases (top). The mutations were selected for heatmap visualization if they were classified as either a predicted driver mutation by the Cancer Genome Interpreter (CGI), annotated as a frequently mutated gene in high-grade meningioma (MNG) tumors, or if the mutation was present in 2 or more tumor samples. The mutations shown in the heatmap are sorted by PyCloneVI clone assignment. The mutations are additionally annotated by consequence type and canonical oncogenic pathway membership.

Next, in order to better understand which, if any, driver mutations besides NF2 may be associated with cancer initiation, progression, and metastases, we used Cancer Genome Interpreter (CGI) to assign known or predicted novel driver status to nonsynonymous mutations. Following this, for clonal evolution analysis, PyCloneVI and ClonEvol were used to infer clonal population structure and clonal ordering from copy number and purity-adjusted VAFs from somatic mutations across all tumor sample timepoints (**Figure 3A**; **Supplementary Figure S4**). Five tumor clones (labeled Cluster/Clone #1 to #5) were detected across the primary meningioma, recurrence, and lung metastases sample timepoints (**Figure 3C**). The primary tumor was composed of two clones: Clone #3 and Clone #5, with Clone #3 present at a clonal prevalence of 0.915. This clone was stably maintained in both the recurrence and lung metastases at clonal prevalence values of 0.676 and 0.968 respectively. Nonsynonymous predicted driver mutations in Clone #3 included NF2 (p.Q389*), as previously described above, BRCA2 (p.E51K), and SEC23B (p.G285V) (**Figure 3B**). This suggests Clone #3 led to the initiation and development of the primary meningioma tumor with NF2 the main driver event. Our analysis also suggests that the presence of BRCA2 may represent a secondary or potentially modifying genetic event; however, this interpretation warrants cautious consideration, particularly given the established role of NF2 mutations in meningioma. Clone #5 was detected only in the primary tumor and contained mutations in genes previously reported to be frequently mutated (> 2 tumors) in high-grade meningioma including LRP1B (7). In addition to Clone #3, the recurrence was composed of two other clones, Clone #1 and Clone #4. Clone #4 contained the driver mutation, previously detailed above, in SETD2 (p.S1885N) and it appears to have not seeded the lung metastases, however, Clone #1 did. The lung metastases were composed of Clone #3, Clone #1, and Clone #2 (**Figures 3D, E**). Although SMARCB1 mutations have previously been shown to be co-mutated in atypical meningiomas (13), we did not identify any SMARCB1 mutations in our study.

Next, having characterized the clonal composition, evolution, and dynamics of the meningioma lung metastases, we used this information along with CGI annotation to identify which mutations were drug response biomarkers (**Table 1**). The only predicted drug-responsive biomarkers detected were NF2 (p.Q389* mutation/copy number loss or deletion) and BRCA2 (p.E51K mutation) in Clone #3, present in all three tumors and SETD2 (p.S1885N mutation) present only in Clone #4 in the recurrence (**Figure 3F**).

**Table 1:** Potential drug-responsive somatic mutations identified using Cancer Genome Interpreter.

Alteration	Pathway	Previously reported in high grade meningioma	Samples	Tumor Clone #	Cancer Type	Drugs	Evidence
NF2 MUT (Q389*)/NF2 Deletion	Hippo	Yes	MUT: M1-T & M3-T SCNA: All	Clone #3	Meningioma	HSP90i; MTORi;AR42 (HDACi);	PMID:23714726; PMID:26015296; PMID:19451225; PMID:2242646; ASCO 2016 (abstr 2558)
BRCA2 MUT (E51K)	Homology-directed DNA Repair	N/A	All	Clone #3	Any cancer type	PARPi; Platinum agent (chemotherapy)	Several Publications; CIVIC; OncoKb; FDA
SETD2 MUT (S1885N)	H3K36me3 Histone modification	Yes	M2-T (Recurrence)	Clone #4	Any cancer type	WEE1 inhibitors	ENA 2014 (abstr 211)

## 3 Discussion

Metastatic meningioma is rare with limited treatment options. Here, molecular-based classification of longitudinal tumors using WGS data, from a case of metastatic meningioma, identified features reported to be characteristic of MG4 proliferative (11, 12) and hypermitotic (12) tumor subtypes. Common characteristics of these tumor molecular subtypes are a higher TMB relative to all other subtypes, risk of recurrence, unfavorable outcomes, and high levels of aneuploidy including copy number loss in chr22q, 1p, and, specific to the MG4 subtype, chr10 loss amongst others (7, 11, 12, 18). Molecular profiling from patients with primary atypical meningiomas, albeit in a small number of patients, has been described previously (19, 20). Within a group of 22 patients profiled by Barresi et al., TMB ranged from 2.19mut/Mb to 12.68mut/Mb at a single timepoint (19). In this case, TMB was assessed in the primary meningioma and the recurrence, showing that the TMB increased over time. Chromosome 1p and 10p loss in particular has been shown to be a strong predictor of decreased recurrence-free survival for patients with WHO grade 2 meningioma following gross total resection (18). In Choudhury et al.*’s* study, the hypermitotic subtype was shown to have decreased immune infiltration compared to an immune-rich subtype (12), suggesting an immunosuppressive tumor microenvironment that may respond to immune checkpoint inhibition. Others have reported that higher-grade meningiomas are composed of several mutations which are predicted to be neoantigens (7). Interestingly, here we identified a stepwise increase in tumor mutational burden from the primary meningioma and recurrence to the lung metastases, suggesting this patient may have been a candidate for immunotherapy (8). Recently, there have been a few phase II clinical trials evaluating the efficacy of anti-programmed death ligand 1 (PD-1) inhibitors in recurrent/progressive grade 2/3 meningioma (21, 22). Immunotherapy response in these trials has been variable, with one trial reporting treatment with nivolumab did not significantly increase the 6-month progression-free survival (PFS-6) (22), while in another, treatment with pembrolizumab did increase PFS-6 (21). However, notably in both trials, there were reports of a long-term durable response to immune checkpoint inhibition in a subset of meningiomas with elevated TMBs, including one patient with distal metastatic disease, treated with pembrolizumab who had a PFS lasting ~20 months. Mismatch repair deficiency (MMRd) is rare in meningioma but was found to be the probable cause of elevated TMB in at least one of the exceptional responder cases (17). Here, MSI testing and mutational signature analysis showed little evidence of MMRd in these tumors. Interestingly, in Bi et al.*’s* study (7), they also observed one case with an elevated level of TMB where there was no evidence for mismatch repair defects, suggesting that the rate of mutagenesis is due to some other mutagenic process. Consistent with this study, we identified a prevalence of C>T transitions, in particular in the recurrence, reported previously in meningioma to be associated with exposure to adjuvant radiation. In the lung metastases, given the smoking status of this patient, the prevalence of C>A mutations are likely due to tobacco-associated mutagenesis.

Clonal evolution analysis confirmed NF2 gene loss as the main driver of this cancer. However, interestingly, in the same cancer clone as NF2, we identified a potential BRCA2 driver mutation (p.E51K) present in all tumors along with a SETD2 (p.S1885N) mutation present only in the recurrence. BRCA-associated protein 1 (BAP1), a tumor suppressor gene whose mutation is often accompanied by NF2 disruption, has previously been described in both *in vitro* and *in vivo* models and is associated not just with meningioma, but also has a significant association with malignant mesothelioma in mouse models (23–26). However, in our study, in both the primary meningioma and lung metastases, the mutational signature analysis indicated some evidence for a mutational signature associated with HRD. Typically, HRD is present in BRCA1/2-mutated or BRCA-*like* tumors and so it could be possible the increase in TMB is due to this type of DNA repair pathway defect, not MMRd. Although not well described in meningioma, BRCA2 mutations are predicted to be a biomarker of drug response to PARP inhibition (27). While our analysis identified a BRCA2 (p.E51K) mutation present alongside NF2 gene loss in the same cancer clone across all tumor samples, it is important to acknowledge that the evidence supporting BRCA2 (p.E51K) as a driver mutation in meningioma is not well-established. Current databases, including ClinVar and OncoKB, classify this variant as being of uncertain biological significance. Furthermore, the absence of this mutation from key somatic cancer mutation references such as COSMIC further emphasizes this ambiguity. The interpretation of BRCA2 (p.E51K) as a driver mutation in this context warrants careful consideration, especially given that NF2 is a well-recognized primary driver in meningiomas. However, the presence of BRCA2 raises the possibility of a secondary role, either as a non-driver mutation or a modifier effect that could impact tumor behavior. While this mutation may represent a potential biomarker of interest, particularly for its predicted responsiveness to PARP inhibitors, definitive conclusions regarding its role and therapeutic implications require further validation.

A SETD2 mutation, which was identified as a predicted biomarker of response to WEE1 inhibition was noted in the recurrence sample (28). Although studies on WEE1 inhibition in meningiomas are limited, preclinical evidence from other brain tumors suggest that WEE1 inhibitors can enhance the effects of radiation and induce tumor cell death (29).

In this patient, for the first- and second-line treatment of their lung metastases, there was a response to sunitinib and bevacizumab for 14 and 4 months respectively. The use of tyrosine kinase inhibition such as sunitinib and monoclonal antibodies targeting anti-angiogenic pathways such as vascular endothelial growth factor (VEGF) signaling has shown antitumoral activity in phase II trials for recurrent meningioma (15, 30, 31). Other tyrosine kinase inhibitors that have also been explored in NF2-associated schwannomas and meningiomas include crizotinib, brigatinib, and dasatinib (32–34). Furthermore, a recent case report demonstrated that it may be possible to use concurrent anti-PD-1 and anti-VEGF in cases of recurrent high-grade metastatic meningioma (35).

There are certain limitations associated with using *in silico* tools, such as CGI, to determine variant significance. While CGI integrates data from multiple databases and algorithms to predict variant impact, not all variants of uncertain significance can be conclusively classified as drivers without further biological validation. Specifically, the classification of the BRCA2 (p.E51K) mutation as a driver remains inconclusive, necessitating additional studies to explore its functional impact in meningiomas. We also noted a high number of structural variants (SVs) in both the primary tumor and lung metastases, however relevant evidence connecting specific SVs to known cancer pathways remains limited.

Despite these limitations, in this case report, molecular characterization and clonal evolution analysis of longitudinal tumors using WGS data identified potentially actionable alterations in meningioma metastases to the lungs. Unfortunately, due to significant clinical deterioration over the months preceding the molecular tumor board, the patient was not administered further lines of systemic anti-cancer treatment. However, this report highlights how clonal evolution analysis and comprehensive genomic alteration profiling can help further our knowledge of this rare entity in cancer but may also guide personalized medicine decision-making.

## Data Availability

The datasets presented in this study can be found in online repositories. The names of the repository/repositories and accession number(s) can be found in the article/**Supplementary Material**.
