# Supplementary material for: Case report: Clonal evolution analysis of a rare case of meningioma lung metastases identifies actionable alterations in matched longitudinal tumour samples
Source: Front Oncol. 2025 Jan 28;14:1483126. doi: 10.3389/fonc.2024.1483126 (PMC11810962; doi:10.3389/fonc.2024.1483126)
Supplement: Supplementary file 1 [file DataSheet1.pdf]

DATA SUPPLEMENT

Whole genome characterization and clonal evolution analysis of a rare case of meningioma lung metastases identifies actionable alterations in matched longitudinal tumor samples

Nicola Cosgrove*, Orla M Fitzpatrick*, Liam Grogan, Bryan T Hennessy, Simon J Furney#, Sinead Toomey#

Table of Contents

Supplementary Materials and Methods p.2

Supplementary Materials and Methods References p.9

Supplementary Figures 1-4 p.10

Supplementary Materials and Methods

Patient consent

This patient provided verbal informed consent, in the presence of his next of kin, for whole genome sequencing.

Sample acquisition

Sufficient tissue for whole genome sequencing (WGS) was available from samples collected from the primary tumour resection (M1-T); the re-excised recurrent tumour (M2-T) after adjuvant radiation therapy; the lung metastases (M3-T) before treatment with Sunitinib and one paired blood sample for tumour-normal analysis.

Sample processing and Whole Genome Sequencing (WGS)

DNA was extracted from tumour samples using the AllPrep DNA mini kit (Qiagen, Hilden, Germany) and from one matched whole blood sample using a DNA blood mini kit (Qiagen), according to the manufacturers protocol. DNA was quantified by Qubit flurometer (Invitrogen, Carlsbad, CA, USA) and DNA integrity examined by agarose gel electrophoresis. For all samples, DNA libraries were sequenced on the BGI Genomics (Hong Kong) DNA Nanoball Sequencing (DNBseq) platform to generate 100bp paired end whole genome sequencing reads.

Sequencing read filtering and sequence alignment

Lane level raw sequencing reads were filtered for adaptor sequence and contamination along with the removal of low quality base pair reads using SOAPnuke[1] software developed by BGI. SOAPnuke software filter parameters: "-n

0.001-l 10 –adaMR 0.25″. If the sequencing read matched 25% or greater of the adapter sequence with a maximum of two base pair mismatches allowed, the entire read was removed. Sequencing reads with 50% or more bases with a quality score less than 10 or were discarded. Also discarded were those reads containing *N* sequences in 0.1% or more of the entire read. Lane level cleaned sequencing reads were aligned separately to the human reference genome (hg38/GRCh38) using the Burrows-Wheeler Aligner (BWA) mem version 0.7.17. According to the GATK4 best practice pipeline (version 4.2.0), read duplicates were marked using Picard MarkDuplicates. For each sample, de-duplicated lane level read alignments were sorted using samtools (v1.9) and merged using MergeSamFiles. Sample level bam files were next processed by base quality score recalibration (BQSR) with the following references supplied with the "--known-sites" option: Homo_sapiens_assembly38.dbsnp138.vcf.gz, Homo_sapiens_assembly38.known_indels.vcf.gz and Mills_and_1000G_gold_standard.indels.hg38.vcf.gz. Prior to somatic variant calling Somalier (v.0.2.13) (https://github.com/brentp/somalier) was used to calculate sample relatedness from sequencing data to ensure normal-tumour sample pairs came from the same individual.

Somatic mutation calling

Somatic single nucleotide variants (SNVs), insertions and deletions (InDels) were called using Strelka (v. 2.9.10)[2] and Mutect2 (v.4.2.0)[3] respectively from matched normal and tumour pairs. In order to improve Strelka's sensitivity for calling InDels >20 nucleotide long, Manta (v.1.6.0) was first used to call structural variants and InDels from mapped paired end sequencing reads. Manta was run using default

settings except for the inclusion of `–callRegions` option to restrict variant calling to chromosomes 1-22, X, Y, M.

Bcftools (v.1.12) (http://samtools.github.io/bcftools/bcftools.html) *norm* function was used to left align and normalise InDels called by Manta. This candidate set of InDels was then used as input to Strelka using `–indelCandidates` along with the inclusion of the `–callRegions` option. In order to filter for false positive somatic mutation calls such as common variants and mapping artifacts, Mutect2 was run with the gnomAD germline population reference and a publicly available panel of normal (PON) sample set provided by the Broad Institute and generated from 1000 Genomes Project samples. FFPE samples are known to contain mutational biases in the C>T / G>A transition. OxoG filter was applied through the read orientation bias model with Mutect2 to remove mutations with FFPE strand bias. GATK4 GetPileupSummaries and CalculateContamination was used with a set number of known germline common variants reported in ExAC at a population minor allele frequency > 0.05 to calculate cross sample contamination. FilterMutectCalls was run using default parameters and passed the output from CalculateContamination (`--contamination-table`), read orientation priors `(--ob-priors)` and stats file from Mutect2 `(--stats)`.

Bcftools *norm* function was used to left align and normalise InDels separately on filtered Mutect2 and Strelka variant call sets followed by GATK4 SelectVariants to select “PASS” only variants. SnpSift annotate and filter was used to filter out common variants in the dbSNP (version 138) database from the left aligned, normalised and filtered variant callsets. Filtered Mutect2 and Strelka somatic variant

calls were combined into one variant call format (VCF) file using GATK3 (v.3.8.1) CombineVariants with `-genotypeMergeOptions` set to PRIORITIZE and the `--rod_priority_list` set to mutect2, strelka. Decomposition of multiallelic sites was performed using *vt* decompose (https://genome.sph.umich.edu/wiki/Vt). Further false positive mutation filtering was applied using the *fpfilter* function, available to use as part of the Varscan mutation calling package. Default settings were used except for `-min-var-count` set to 3 and `-min-var-freq` 0.01. Variant annotation was performed using variant effect predictor (VEP v.96; GRCh38 reference genome). The VCF generated by VEP was used as input to the Memorial Sloan Kettering cancer centre (MSKCC) developed tool *vcf2maf* (https://github.com/mskcc/vcf2maf) to convert the VCF to maf file format. This maf file was used as input to the MSKCC *ngs-filters* (https://github.com/mskcc/ngs-filters) tool to flag and filter low confidence mutations (alternative (ALT) allele Count > 1 in the normal sample or < 4 in the tumour sample or tumour sequencing DEPTH <= 10) and any mutations flagged as a potential FFPE artifact. Only those annotated filtered variants called by both Mutect2 and Strelka i.e., labelled “intersection set” were imported into the R statistical programming environment using MAFTools[4] for downstream somatic mutation analysis.

Estimation of tumour mutational burden

Tumour mutational burden (TMB) is defined here as the number of nonsynonymous somatic mutations per megabase of exome. The nonsynonymous mutation rate per Mb was calculated used MAFTools as the total number of coding variants (SNVs, indels) divided by the exome total length in megabases (50Mb).

Annotation of driver mutations and drug response biomarkers

An online platform Cancer Genome Interpreter[5] (CGI) was utilised for assigning oncogenic driver (predicted / known) or passenger mutation status to each gene alteration. CGI was also used for identifying which alterations may be targetable in meningioma and if they were likely to be a marker of drug response/resistance. A detailed description of the CGI implementation is available at https://www.cancergenomeinterpreter.org/faq. Briefly, given a list of genomic alterations for a specific tumour type, CGI implements machine learning-based methods called boostDM and OncodriveMut for in-silico saturation mutagenesis of cancer genes to assess the oncogenic potential of mutations in human tissues. Oncogenic potential prediction is given from machine learning models trained on mutational features of cancer genes such as the functional consequence type and positional clustering of the mutation. CGI also cross-references mutations against a catalogue of validated oncogenic mutations, compiled from multiple databases containing cancer mutation knowledge, including ClinVar, OncoKB and DoCM. Furthermore, to assess the relevance of the alterations as biomarkers of drug response, CGI performs annotation of inputted genomic alterations using an in-house curated database called Cancer Biomarkers and two other publicly available resources: CIViC and OncoKB. Annotation of biomarker drug response is given according to different levels of therapeutic evidence such as clinical evidence (already an FDA-recognised biomarker predictive of response to an FDA-approved drug in this indication), guidelines (standard of care biomarker of response / resistance), compelling clinical and/or biological evidence from early clinical trials, case reports or pre-clinical testing.

Mutational Signatures

Somatic point mutations were used as input for mutational signature analysis using the *signature.tools.lib* (version 2.4.1) R package, developed by the Nik Zainal Group, which implements the Signal[6] framework for mutational signature extraction and signature fitting. Single base substitutions were categorised by their trinucleotide context to generate a 96-channel mutational, SNV catalogue matrix for each sample. Regions of clustered substitutions i.e., kaetegis regions were filtered if present. The SignatureFit algorithm was used with bootstrapping to fit COSMIC30 reference mutational signatures (v2 - March 2015) to the SNV catalogue matrices using the *Fit* function with the following parameters: 100 bootstraps, method = "KLD", threshold p-value < 0.05. Signature exposure values correspond to the median of the bootstrapped runs.

Somatic copy number calling

Allele specific DNA copy number inference using FACETS

Total and allele-specific copy number states were inferred for all tumour samples using FACETS Suite (v 2.0.8) and FACETS (v.0.6.1) (https://github.com/mskcc/facets-suite). Tumour and matched normal bam files were pre-processed using snp-pileup (v.0.6.1) with parameters –q15 –Q20 –P100 –r25,0. A two pass implementation of FACETS using snp pileup file output as input, was utilised were a low sensitivity run (cval =100) first infers the purity and log-ratio related to diploidy, as per methodology[7]. A second higher sensitivity run (cval=50) to detect focal events, determines the copy number state of each gene.

Somatic Structural Variant (SV) Calling

DELLY2[8] was used to call structural variants (SVs) (deletions, duplications, inversions and translocations) from WGS sequence alignment data (min mapping quality 20) from each tumour-normal sample pair. Filtering was applied to DELLY2 output to select only somatic SVs with a minimum 500bp length, a 20X minimum tumour coverage with minimum allele frequency cut off of 0.01. BCFTools was used to select “PASS” only SV’s, classified as “PRECISE” with a split read support of greater than or equal to 5. AnnotSV (https://github.com/lgmgeo/AnnotSV) was used to annotate filtered somatic SVs with the StructuralVariantAnnotation and circlize R packages used for circos plot visualisation.

Clonal evolution analysis

PyClone-VI (v.0.1.1)[9] was used to infer the clonal population structure within longitudinally collected matched tumour samples. To prepare PyClone-VI compatible input files, filtered PASS only mutations from each tumour sample were concatenated together use bcftools merge to generate a “master” VCF to guide force counting of REF and ALT alleles using *GetBaseCountsMultiSample* (https://github.com/zengzheng123/GetBaseCountsMultiSample) across all sequence alignment bam files from the tumour and normal samples. Variant allele frequency data was integrated with allele specific copy number calls and tumour purity values from FACETS using FACETS Suite based on the McGranahan *et al.,* methodology[10] for estimating the cancer cell fraction (CCF) for each mutation. Copy number and purity adjusted mutations with a major copy number >0 were clustered using PyClone-VI with the following parameters: maximum of 40 clusters, using the beta binomial probability density distribution for allele counts, performing 10 random

restarts with 10,000 max iterations. Clonal prevalence was calculated at each time point by taking the median cellular prevalence value for each mutation cluster (clone). PyClone-VI output was used as input to ClonEvol[11] to infer clonal population structure, clonal ordering and visualisation. fishplot[12] was used to visualise the changes in the clonal architecture of the tumours over time.

Supplementary Materials and Methods References

1. Chen, Y. *et al.* SOAPnuke: a MapReduce acceleration-supported software for integrated quality control and preprocessing of high-throughput sequencing data. *Gigascience* **7**, 1-6 (2018).
2. Saunders, C.T. *et al.* Strelka: accurate somatic small-variant calling from sequenced tumor-normal sample pairs. *Bioinformatics* **28**, 1811-7 (2012).
3. Benjamin, D. *et al.* Calling Somatic SNVs and Indels with Mutect2. (Cold Spring Harbor Laboratory, 2019).
4. Mayakonda, A., Lin, D.C., Assenov, Y., Plass, C. & Koeffler, H.P. Maftools: efficient and comprehensive analysis of somatic variants in cancer. *Genome Res* **28**, 1747-1756 (2018).
5. Tamborero, D. *et al.* Cancer Genome Interpreter annotates the biological and clinical relevance of tumor alterations. *Genome Med* **10**, 25 (2018).
6. Degasperi, A. *et al.* Substitution mutational signatures in whole-genome–sequenced cancers in the UK population. *Science* **376**(2022).
7. Bielski, C.M. *et al.* Widespread Selection for Oncogenic Mutant Allele Imbalance in Cancer. *Cancer Cell* **34**, 852-862 e4 (2018).
8. Rausch, T. *et al.* DELLY: structural variant discovery by integrated paired-end and split-read analysis. *Bioinformatics* **28**, i333-i339 (2012).
9. Gillis, S. & Roth, A. PyClone-VI: scalable inference of clonal population structures using whole genome data. *BMC Bioinformatics* **21**(2020).
10. McGranahan, N. *et al.* Clonal status of actionable driver events and the timing of mutational processes in cancer evolution. *Sci Transl Med* **7**, 283ra54 (2015).
11. Dang, H.X. *et al.* ClonEvol: clonal ordering and visualization in cancer sequencing. *Ann Oncol* **28**, 3076-3082 (2017).
12. Miller, C.A. *et al.* Visualizing tumor evolution with the fishplot package for R. *BMC Genomics* **17**, 880 (2016).

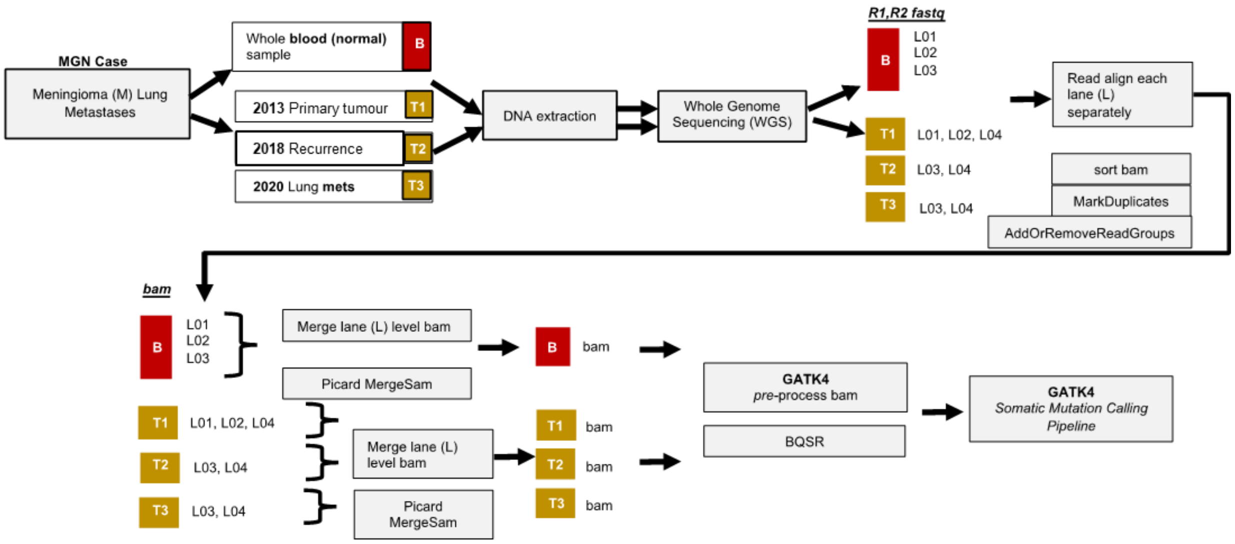

Fig S1. Overview of whole genome sequencing (WGS) data processing workflow. Graphical overview of whole genome sequencing data processing workflow for matched blood normal (red box) and tumour samples: primary meningioma tumour (T1), primary tumour recurrence (T2), lung metastases (T3) (mustard). Each sample was sequenced across different sequencing lanes (L).

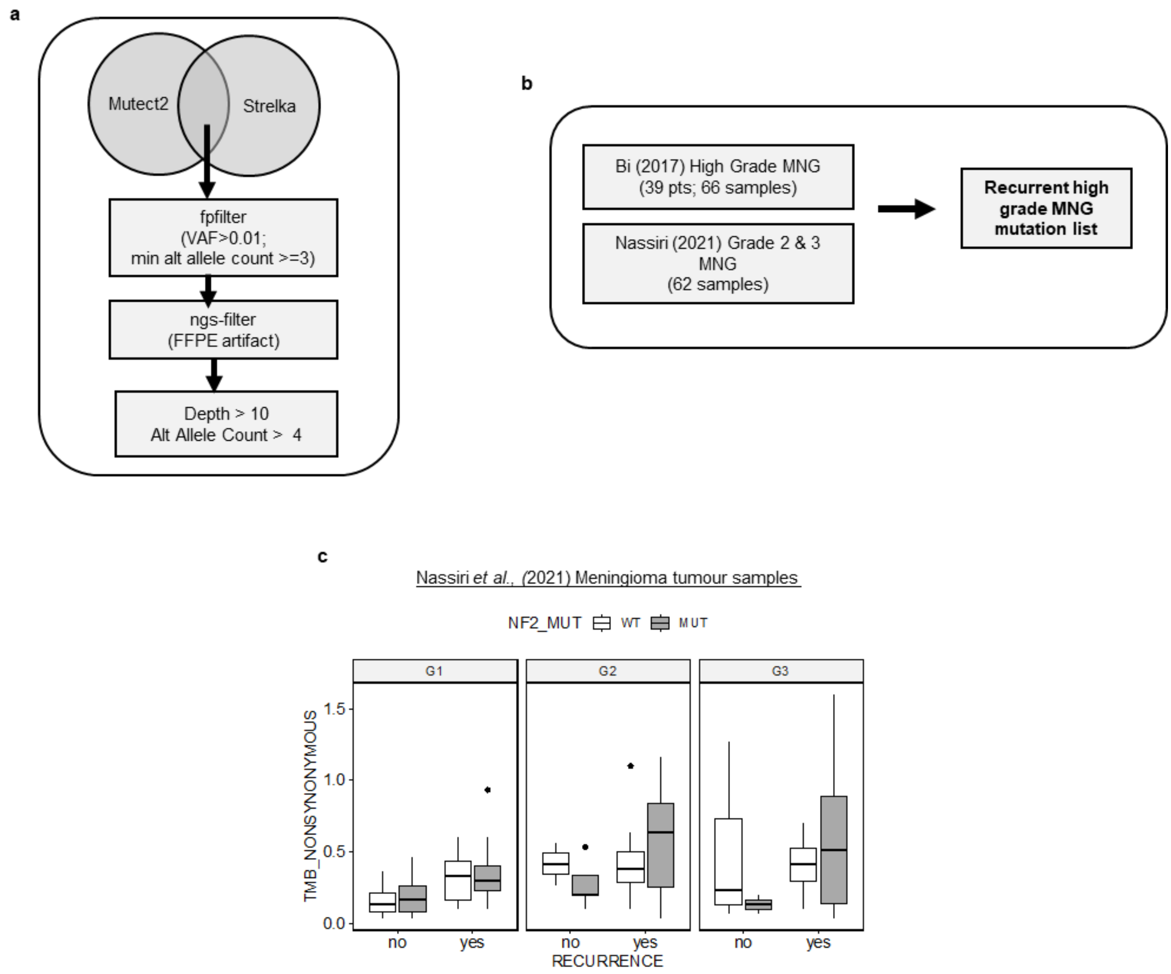

Fig S2. Somatic mutation data processing and TMB estimates in high grade meningioma tumours. (a) Graphical overview of filtering applied to somatic mutation calls from Mutect2 and Strelka intersection set. VAF: variant allele frequency **(b)** Graphical overview of the publicly available data sources utilized for generation of a reference list of recurrent gene mutations in high grade, Grade 2 or Grade 3 meningioma (MNG) tumour samples **(c)** Boxplots of tumour mutational burden (TMB) (nonsynonymous mutations per megabase (Mb)) estimates from the Nassiri et al., (2021) meningioma cohort (n=121 samples). TMB estimates from meningiomas stratified by WHO Grade tumour classification, tumour recurrence status and NF2 gene mutations status (MUT=mutated (white), WT=wildtype (grey)).

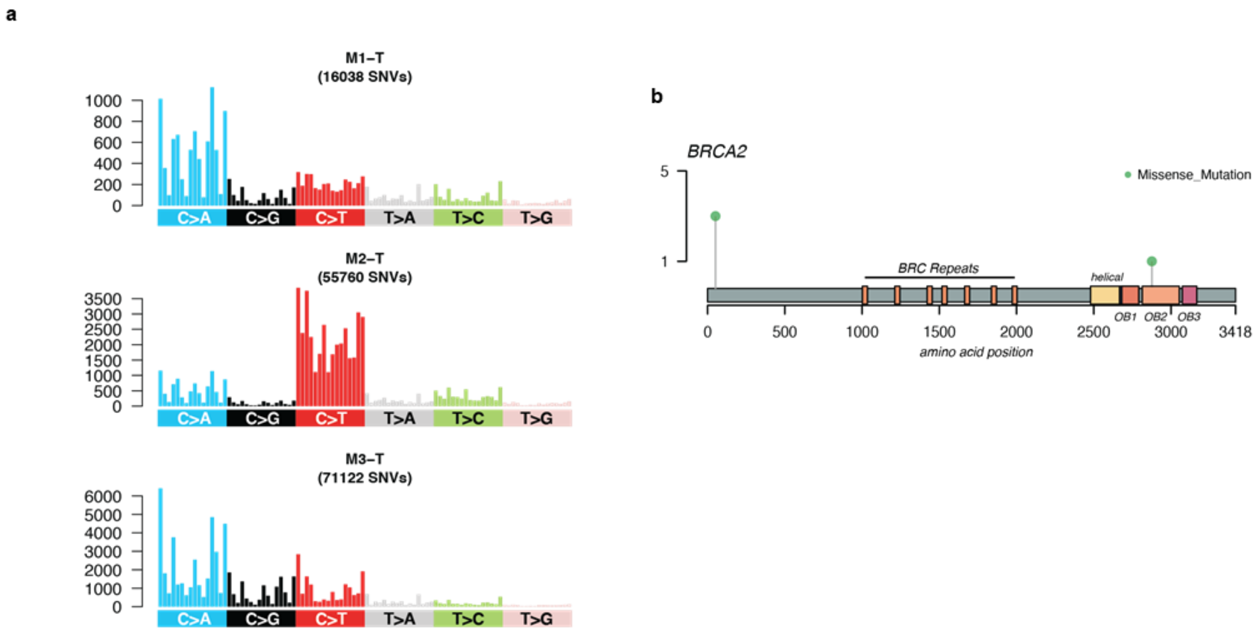

Fig S3. Patterns of single nucleotide mutation and BRCA2 mutation overview (a) Barchart of six channel mutation context profile counts for all tumour samples **(b)** Lolliplot of BRCA2 gene mutations.

a

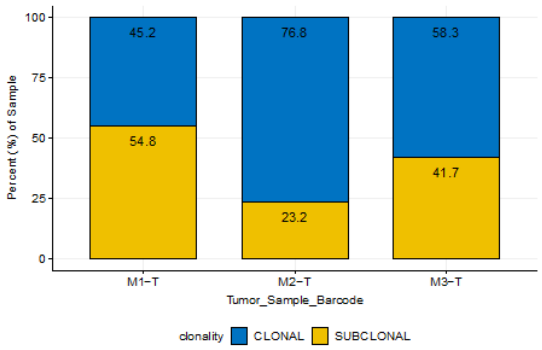

b

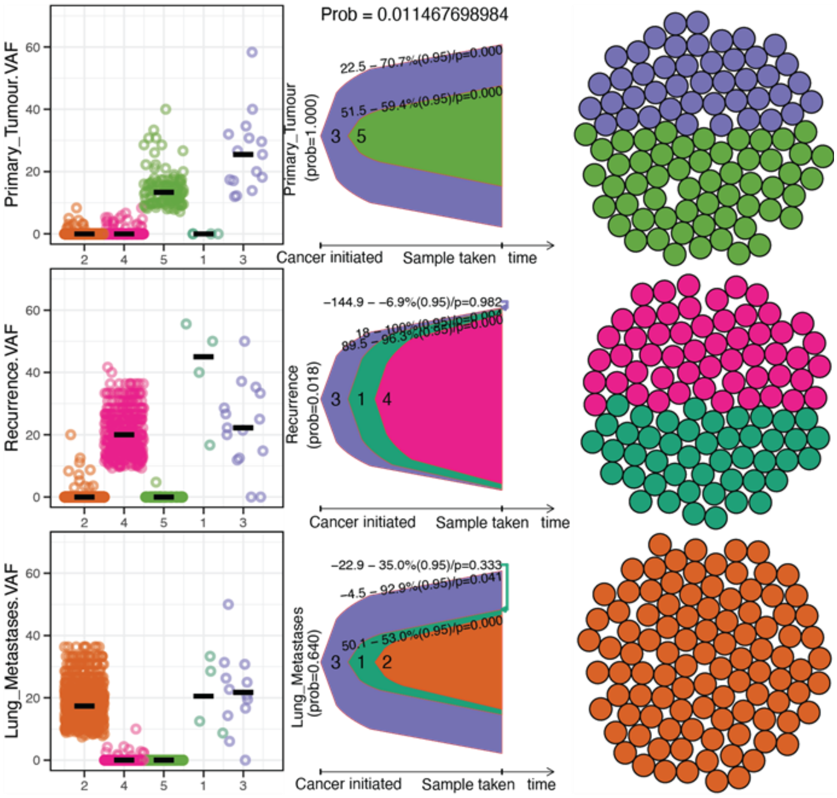

Fig S4. Clonal mutation status and clonal ordering and visualization using ClonEvol. (a) Stacked barchart shows the percent (%) clonal (blue) and subclonal (yellow) somatic mutations for each tumour sample (left to right) **(b)** Boxplots (left panel) of variant allele frequency (VAF) values across PyCloneVI derived tumour clones (Clone #1 (dark green), #2 (orange), #3 (purple), #4 (pink), #5 (lime green)). Middle and right panel shows bell plots and "sphere of cell" plots from Clonevol clonal ordering visualization output.
